# Medication risks in older patients (70 +) with cancer and their association with therapy-related toxicity

**DOI:** 10.1186/s12877-022-03390-z

**Published:** 2022-08-30

**Authors:** Imke Ortland, Monique Mendel Ott, Michael Kowar, Christoph Sippel, Yon-Dschun Ko, Andreas H. Jacobs, Ulrich Jaehde

**Affiliations:** 1 Institute of Pharmacy, Department of Clinical Pharmacy, University of Bonn, An der Immenburg 4, 53121 Bonn, Germany; 2 Department of Geriatrics and Neurology, Johanniter Hospital Bonn, Johanniterstr. 1-3, 53113 Bonn, Germany; 3 Department of Oncology and Hematology, Johanniter Hospital Bonn, Johanniterstr. 1-3, 53113 Bonn, Germany

**Keywords:** Polymedication, Potentially inappropriate medication, Drug-drug interactions, Older patients with cancer, Toxicity, Onco-geriatrics

## Abstract

**Background:**

To evaluate medication-related risks in older patients with cancer and their association with severe toxicity during antineoplastic therapy.

**Methods:**

This is a secondary analysis of two prospective, single-center observational studies which included patients ≥ 70 years with cancer. The patients’ medication lists were investigated regarding possible risks: polymedication (defined as the use of ≥ 5 drugs), potentially inappropriate medication (PIM), and relevant potential drug-drug interactions (rPDDI). The risks were analyzed before and after start of cancer therapy. Severe toxicity during antineoplastic therapy was captured from medical records according to the Common Terminology Criteria for Adverse Events (CTCAE). The association between grade ≥ 3 toxicity and medication risks was evaluated by univariate as well as multivariate regression adjusted by ECOG and age.

**Results:**

The study cohort comprised 136 patients (50% female, mean age 77 years, 42% hematological malignancies). Before the start of cancer therapy, patients took on average 5 drugs as long-term medication and 52% of patients were exposed to polymedication. More than half of patients used at least one PIM. Approximately one third of patients exhibited rPDDI. The prevalence of medication risks increased after start of cancer therapy. rPDDI were significantly associated with severe overall toxicity (OR, 5.07; *p* = 0.036; 95% Confidence Interval (CI) 1.11–23.14; toxicity in patients with rPDDI 94.1% (32/34) vs 75.9% (60/79) in patients without rPDDI) and hematological toxicity (OR, 3.95; *p* = 0.010; 95% CI 1.38–11.29; hematological toxicity in patients with rPDDI 85.3% (29/34) vs 59.5% (47/79) in patients without rPDDI). In the multivariate analysis adjusted by ECOG and age, only the association for rPDDI with hematological toxicity remained statistically significant (OR, 4.51; *p* = 0.007; 95% CI 1.52–13.38). These findings should be further investigated in larger studies.

**Conclusion:**

Medication risks are common in older patients with cancer and might be associated with toxicity. This raises the need for tailored interventions to ensure medication safety in this patient cohort.

**Supplementary Information:**

The online version contains supplementary material available at 10.1186/s12877-022-03390-z.

## Background

Drug-related problems are an important issue affecting patient safety in older cancer patients. Elderly cancer patients show a higher risk because of altered pharmacokinetics/-dynamics, a higher prevalence of concomitant chronic diseases, and a higher drug burden. A retrospective analysis showed that 90% of older patients with cancer exhibited drug-related problems (DRP) [1]. A medication review for older cancer patients is therefore recommended and regarded as an essential aspect of the geriatric assessment [2, 3]. There are in particular three aspects of drug-related problems which play an important role in older cancer patients: polymedication, potentially inappropriate medication, and drug-drug interactions. A study with 385 older cancer patients observed polymedication in 57% of patients [4]. Also, polymedication was associated with adverse events like falls and chemotherapy toxicity in older cancer patients [5]. However, when judging the quality of their medication, it is not only important to consider *how many* drugs, but also *which* drugs are used. “Potentially inappropriate medications (PIM)” are drugs where risks may outweigh benefits in older patients. A study with 160 older patients receiving parenteral cancer therapy in an ambulatory clinic indicated that 48.1% used at least one PIM [6]. PIM drugs were associated with adverse outcomes like postoperative complications, higher mortality, and decreased progression-free survival [5]. Moreover, potential drug-drug interactions are frequent among older patients with cancer. Yeoh et al. detected potential drug-drug interactions as the most frequent drug-related problem (36.4%) in older patients receiving outpatient chemotherapy [1]. Clinical consequences of those interactions might be serious, even leading to unplanned hospitalizations in some cases [7].

The aim of our study was (I) to assess the medication risks of older patients with cancer regarding polymedication, potentially inappropriate medication as well as drug-drug interactions before and after start of cancer therapy, and (II) to analyze their association with toxicity for evaluating their clinical impact. This study fills a gap in knowledge being the first study assessing medication risks of elderly cancer patients in a German hospital setting.

## Methods

### Study design and procedures

This was a secondary analysis of the medication data from two prospective, single-center observational studies, namely a pilot study (*n* = 20) and the respective evaluation study (*n* = 120) concerning the prediction performance of the CARG (Cancer and Aging Research Group) and the CRASH (Chemotherapy Risk Assessment Scale for High-Age Patients) score. Those results have already been described elsewhere [8]. A positive vote of the ethics committee of the Faculty of Medicine of the University of Bonn was granted for both studies and all patients signed an informed consent. The studies took place from March to June 2015 and November 2015 to August 2017. Patients were recruited at the inpatient wards of the Johanniter Hospital Bonn with the following inclusion criteria: ≥ 70 years, diagnosis of a malignancy, German language skills, and scheduled to start inpatient first-line systemic cancer therapy. Exclusion criteria were moderate to severe cognitive impairment (Mini-Mental State Examination < 20) or previously started cancer therapy. Only those patients who were actually treated with a systemic cancer treatment were included because not all patients of the pilot study received a systemic cancer therapy later on. The medication was captured from medical records and analyzed at two time points. First, the medication was investigated at the time of admission to the hospital for assessing the risks of long-term medication which patients received before the start of treatment. Second, after the start of cancer treatment, the medication was analyzed including antineoplastic agents and supportive care during the first treatment cycle for investigating the risks during cancer therapy.

### Medication

In general, the medication was counted per active ingredient and not per medicinal product. All active ingredients with systemic effects were collected and classified according to the Anatomical Therapeutic Chemical Classification (ATC code), level 2 (therapeutic subgroups) [9]. Herbal medication was included whereas dietary supplements, medical devices, electrolyte solutions or medical gases were not considered. Because the focus on this analysis was on long-term medication, all paused drugs and all drugs just being started at the day of admission or just used in case of acute symptoms were excluded. Concerning antineoplastic agents and supportive care medication, all drugs reported on the therapy plan of the first cycle were included. For enhancing the clinical relevance of findings, the supportive care medication was only considered as PIM or regarding drug-drug interactions if it was applied more than once during hospital stay.

#### Polymedication

In this analysis, polymedication was defined as the concomitant use of ≥ 5 drugs. This cut-off value is commonly used and has been associated with adverse outcome in the elderly [10]. Excessive polymedication (“hyperpolymedication”) was defined as the use of ≥ 10 drugs as discussed by Sharma et al. [10].

#### Potentially inappropriate medication

The EU(7)-PIM list was used, an explicit PIM list which is widely applicable across Europe and is based on the German PRISCUS list [11]. According to the EU(7)-PIM list, some drugs are only regarded as PIM under certain conditions. In particular, proton-pump inhibitors (PPI) are only classified as PIM if taken longer than eight weeks [11]. If the duration of drug use was unknown, this study classified PPI as PIM unless any evidence was found that the PPI was applied for < 8 weeks.

#### Relevant potential drug-drug interactions

Drug-drug interactions were classified according to the ABDA (Federal Union of German Associations of Pharmacists) interaction database which is the most commonly used interaction database in German community pharmacies [12]. Because further clinical information was missing, all observed drug-drug interactions were assumed to be potential. For enhancing the clinical relevance of findings, this analysis particularly focused on severe potential drug-drug interactions. These “relevant potential drug-drug interactions” (rPDDI) only included those five ABDA classifications which require an intervention or action by health care providers (“Serious consequences possible – contraindicated”; “serious consequences possible – in certain cases contraindicated”; “serious consequences possible – as precaution contraindicated”; “simultaneous usage not recommended”, or “monitoring/modification required”). Regarding cancer therapy, all interactions between the antineoplastic agents and supportive care medication were excluded because those specific combinations have been established in clinical protocols and are widely used in daily routine. “Desired” rPDDI (e.g. methotrexate and folic acid) were not taken into account either. For determining the risk of a drug class being involved as an interaction partner in rPDDI, a prevalence-adjusted ratio was calculated which will be referred to as “interaction propensity”, see Eq. 1:

1 $$IP=\frac{{F}_{i}}{{P}_{d}}$$

IP = Interaction propensity.

F_i_ = Relative frequency of a drug or drug class being involved as interaction partner in rPDDI.

P_d_ = Prevalence of a drug or drug class.

### Toxicity assessment

The incidence of severe toxicity during the therapy course was captured via a standardized form from medical records according to the Common Terminology Criteria for Adverse Events (CTCAE), version 4.03 [13]. Severe toxicity was defined as CTCAE grade 3 (hospitalization indicated), grade 4 (life-threatening) or grade 5 (treatment-related death). Patients were observed until the end of antineoplastic therapy or for a maximum of six cycles [8].

### Statistical analysis

Descriptive analyses were carried out for medication data. Furthermore, a univariate logistic regression was performed for determining whether risks in long-term medication before start of cancer treatment were associated with overall, hematological, and nonhematological toxicity. Associations detected in the univariate analysis were further analyzed by adjusting with the potential confounders ECOG and age in a multivariate analysis. Polymedication, PIM, and rPDDI were treated as continuous as well as categorial variables. Analyses were performed using Microsoft® Excel® 2007 (Microsoft Corporation, Redmond, USA) and IBM® SPSS® Statistics Version 25.0 for Windows (IBM Corporation, Armonk, USA). A *p*-value of < 0.05 was considered statistically significant and 95% confidence intervals were computed.

## Results

### Patient characteristics

**Fig. 1:**
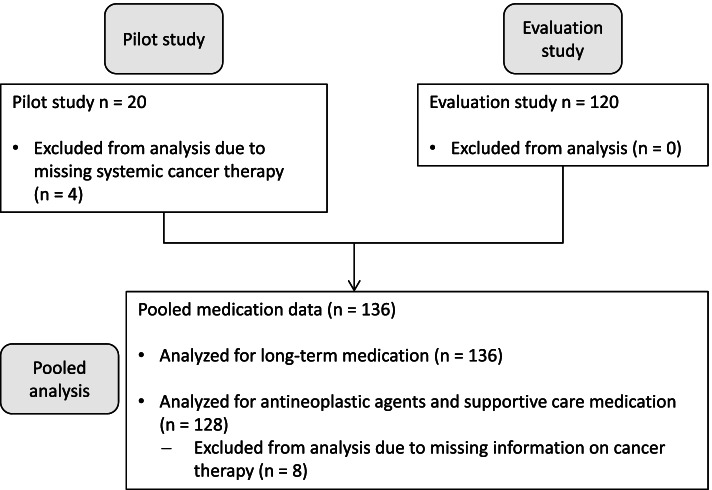
Flow chart of patient inclusion for medication risk analysis

In total, 136 patients were included from the respective studies. A flow chart of the patient inclusion is given in Fig. 1. The patient characteristics are displayed in Table 1.

**Table 1:** Characteristics of the patients included into the medication risk analysis (*n* = 136)

**Age [years]**		
Mean (SD)	76.9 (4.53)	
Range	70–88	
**Charlson Comorbidity Index**^**a**^		
Mean (SD)	1.05 (1.237)	
Range	0–7	
**Creatinine Clearance (Cockcroft-Gault) [mL/min]**		
Mean (SD)	67.2 (22.89)	
Range	10–131	
	**n**	**%**
**Sex**		
Female	68	50.0
Male	68	50.0
**ECOG performance status**		
Fully active (0)	45	33.1
Capable of all self-care (1–2)	74	54.4
Limited or no self-care (3–4)	17	12.5
**Tumor entity**^**b**^		
Respiratory	34	25.0
Hematological	57	41.9
Gynecological	5	3.7
Genitourinary	3	2.2
Unknown primary	4	2.9
Musculoskeletal	1	0.7
Digestive/gastrointestinal	16	11.8
Breast	13	9.6
Others	3	2.1
**Relapse**		
No	118	86.8
Yes	18	13.2
**Cancer stage**		
I	7	5.1
II	11	8.1
III	31	22.8
IV	68	50.0
Missing	19	14.0
**Treatment type**		
Chemotherapy	81	59.6
Targeted or immunotherapy	9	6.6
Combined chemotherapy and targeted or immunotherapy	46	33.8

*ECOG* Eastern Cooperative Oncology Group

^a^ Considering conditions in addition to primary cancer diagnosis

^b^ By body location according to the National Cancer Institute (NCI)

### Long-term medication before start of cancer therapy

Almost all patients used long-term medication before the start of cancer therapy, only 8/136 (5.9%) patients did not take any regular long-term medication at time of admission. On average, patients took 5 drugs (standard deviation (SD), 3.5). Most drugs were only available on prescription (587/683); solely 96/683 drugs comprised over-the-counter (OTC) drugs. The most frequently used drug classes were antithrombotic agents (ATC Code B01; mostly acetylsalicylic acid, ASS), agents acting on the renin-angiotensin system (ATC Code C09; mostly ramipril), and diuretics (ATC Code C03; mostly hydrochlorothiazide). Regarding the active ingredients, pantoprazole, L-thyroxine, and ASS were the most frequently used drugs. Drug classes and individual drugs of patients’ long-term medication are given in Supplement 1.

#### Polymedication

More than half of patients (71/136, 52.2%) exhibited polymedication (≥ 5 drugs) and approximately every 10^th^ patient (14/136, 10.3%) was exposed to hyperpolymedication (≥ 10 drugs).

#### Potentially inappropriate medication

Patients took in median one ( interquartile range (IQR), 1; range, 0–5) PIM drug. More than half of the patients (72/136, 52.9%) used at least one PIM drug before start of cancer therapy. By far the most frequent PIM drugs were drugs for acid-related disorders (ATC A02). Consistent with this finding, pantoprazole was the most frequently taken PIM drug (42/136 patients). Other commonly used PIM drug classes comprised drugs used in diabetes (ATC code A10; mostly sitagliptin), drugs for cardiac therapy (ATC code C01; mostly amiodarone), and calcium channel blockers (ATC code C08; mostly verapamil). An overview of the individual PIM drugs and the respective drug classes is presented in Table 2.

**Table 2:** Prevalence of PIM drugs in long-term medication before start of cancer therapy (*n* = 136)

**PIM drug (ATC code)**	**Number of patients**	**Proportion of patients with respective drug [%]**
Pantoprazole (A02BC02)	42	30.9
Sitagliptin (A10BH01)	8	5.9
Amiodarone (C01BD01)	4	2.9
Verapamil (C08DA01)	4	2.9
Rivaroxaban (B01AF01)	3	2.2
Omeprazole (A02BC01)	3	2.2
Amitriptyline (N06AA09)	3	2.2
Sotalol (C07AA07)	3	2.2
Diclofenac (M01AB05)	2	1.5
Diltiazem (C08DB01)	2	1.5
Methocarbamol (M03BA03)	2	1.5
Metoclopramide (A03FA01)	2	1.5
Pramipexole (N04BC05)	2	1.5
Trospium (G04BD09)	2	1.5
**PIM drug class (ATC code level 2)**	**Number of drug prescriptions**	
Drugs for acid-related disorders (A02)	47	
Drugs used in diabetes (A10)	10	
Cardiac therapy (C01)	8	
Calcium channel blockers (C08)	7	
Psycholeptics (N05)	6	
Psychoanaleptics (N06)	5	
Antithrombotic agents (B01)	4	
Anti-inflammatory and antirheumatic products (M01)	3	
Beta blocking agents (C07)	3	
Urologicals (G04)	3	
Others	11	

#### Relevant potential drug-drug interactions

Approximately one third of the patients (42/136, 30.9%) exhibited relevant potential drug-drug interactions (rPDDI) in long-term medication before the start of cancer therapy. The majority of rPDDI was classified as “monitoring/modification required” (67/71 rPDDI) according to the ABDA database. Only 4/71 rPDDI were categorized as “simultaneous usage not recommended”. No contraindications were observed. Most rPDDI were pharmacodynamic interactions (40/71 rPDDI) whereas 21/71 rPDDI were due to altered pharmacokinetic processes. In general, a variety of interaction types was observed. The most frequent rPDDI comprised “anti-diabetic drugs – corticosteroids”, “agents acting on the renin-angiotensin system – heparinoids” and “simvastatin – amlodipine”. Frequently detected rPDDI are presented in Table 3.

**Table 3:** rPDDI before start of cancer therapy (*n* = 136) and after start of cancer therapy (*n* = 128)

**Type of interaction**	**Number of interactions**	**ABDA database classification**	**Mechanism/effect of interaction**
**Interactions before start of cancer therapy**			
Anti-diabetic drugs – corticosteroids	8	Monitoring/modification needed	Hyperglycemic effect of corticosteroids
Agents acting on the renin-angiotensin system – heparinoids	8	Monitoring/modification needed	Increased risk of hyperkalemia
Simvastatin – amlodipine	8	Monitoring/modification needed	Amlodipine inhibits simvastatin metabolism via CYP3A4 leading to higher risk of myopathy
Beta agonists – beta blocker	6	Monitoring/modification needed	Antagonistic effects
ACE inhibitors – allopurinol	5	Monitoring/modification needed	Increased risk of immunologic reactions (mechanism unknown)
Amiodarone – beta blockers	4	Monitoring/modification needed	Additive cardio depressive effects
Thyroid hormones – polyvalent cations	4	Monitoring/modification needed	Decreased effect of thyroid hormones due to reduced resorption
Insulins – cardio selective beta blockers	3	Monitoring/modification needed	Increased risk of hypoglycemia, masking of hypoglycemic symptoms
NSAIDs – corticosteroids	3	Monitoring/modification needed	Higher risk of gastrointestinal ulcer
Thiazide-diuretics – vitamin D	3	Monitoring/modification needed	Higher risk of hypercalcemia
Others	19	Various	Various
**Interactions after start of cancer therapy**			
NSAIDs – corticosteroids	8	Monitoring/modification needed	Higher risk of gastrointestinal ulcer
Cytotoxic agents – thiazide diuretics	7	Monitoring/modification needed	Increased myelosuppressive effects
Anti-diabetic drugs – corticosteroids	5	Monitoring/modification needed	Hyperglycemic effect of corticosteroids
ACE inhibitors – allopurinol	4	Monitoring/modification needed	Increased risk of immunological reactions (mechanism unknown)
Hyperkalemic drugs – trimethoprim	4	Monitoring/modification needed	Increased risk of hyperkalemia due to additive effects on potassium levels
QT prolonging drugs – antidepressant	3	Simultaneous usage not recommended	Increased risk of torsades de pointes
QT prolonging drugs – antiarrhythmic agent	3	Serious consequences possible – as precaution contraindicated	Increased risk of torsades de pointes
Loop diuretics – platinum compounds	3	Monitoring/modification needed	Higher risk of nephrotoxicity/ototoxicity
Nitrogen mustard derivatives – allopurinol	3	Monitoring/modification needed	Additive myelotoxic effects
Fluoropyrimidines – folate ^a^	2	Monitoring/modification needed	Higher toxicity of fluoropyrimidines
Others	8	Various	Various

*ABDA* Federal Union of German Associations of Pharmacists, *NSAIDs* Nonsteroidal anti-inflammatory drugs

^a^ Interaction was unintended in this case

The most frequent drug classes being involved in rPDDI were agents acting on the renin-angiotensin system (ATC code C09), beta blocking agents (ATC code C07), and antithrombotic agents (ATC code B01); see Table 4. According to the interaction propensity, the drug classes with the highest probability of provoking interactions were cardiac therapy (ATC code C01) and corticosteroids for systemic use (ATC code H02). Although being the drug class most frequently involved in interactions regarding absolute numbers, agents acting on the renin-angiotensin system did not show such a high risk of provoking rPDDI after being adjusted for prevalence.

**Table 4:** Frequency of drug classes in long-term medication being involved in rPDDI and the respective interaction propensity; *n* = 136

Drug class (ATC code level 2)	Number of detected interactions	Interaction propensity
Agents acting on the renin-angiotensin system (C09)	17	0.26
Beta blocking agents (C07)	13	0.26
Antithrombotic agents (B01)	13	0.19
Corticosteroids for systemic use (H02)	12	0.75
Drugs used in diabetes (A10)	12	0.41
Lipid modifying agents (C10)	11	0.23
Diuretics (C03)	9	0.15
Cardiac therapy (C01)	9	1.0
Calcium channel blockers (C08)	8	0.27
Drugs for obstructive airway diseases (R03)	6	0.25
Others	32	-

### Antineoplastic agents and supportive care medication

In total, the medication of 128 patients could be assessed after initiation of cancer therapy. The patients received in median 6 (IQR, 2.25; range, 1–12) additional drugs. This comprised in median 2 (IQR, 1; range, 1–5) additional drugs for antineoplastic therapy and in median 4 (IQR, 2.25; range, 0–7) additional drugs for supportive therapy. Regarding antineoplastic agents, “plant alkaloids and other natural products” (ATC code L01C, e.g. paclitaxel) as well as “platinum compounds” (ATC code L01XA, e.g. carboplatin) were the most frequently prescribed drug classes. Concerning supportive care medication, the most frequently used drug class by far was “antiemetics and antinauseants” (ATC code A04, e.g. ondansetron). Details regarding the prevalence of antineoplastic agents and supportive care medication are presented in Supplement 2.

#### Potentially inappropriate medication

After the start of cancer therapy, 36.7% (47/128) of patients received further PIM drugs being used more than once per cycle. The most commonly used additional PIM drug was ranitidine (32/128), followed by clemastine (17/128) and proton-pump inhibitors (8/128).

#### Relevant potential drug-drug interactions

After the start of cancer therapy, 29.7% (38/128) of patients demonstrated further rPDDI between the long-term medication and the antineoplastic agents/supportive care medication. The interaction types were diverse. The most frequently observed rPDDI comprised “NSAIDs – corticosteroids” and “cytotoxic agents – thiazide diuretics”, as well as “anti-diabetic drugs – corticosteroids” (see Table 3). The rPDDI were usually categorized as “monitoring/modification required” by the ABDA database classification. Three out of hundred twenty-eight (2.3%) patients exhibited contraindications. However, no patient exhibited more than one contraindication. The most severe interaction types involved QT prolonging agents. Most rPDDI consisted of pharmacodynamic interactions (30/50 rPDDI). Changes in pharmacokinetics only rarely caused rPDDI (5/50 rPDDI). “Corticosteroids for systemic use” (ATC code H02) was the drug class most frequently causing rPDDI. However, “antibiotics” (ATC code J01) showed the highest interaction propensity (0.86). This was triggered by the numerous interactions between trimethoprim and ACE inhibitors. Respective details are provided in Table 5.

**Table 5:** Drug classes being involved in rPDDI between antineoplastic agents/supportive care medication and the long-term medication; *n* = 128

Drug class (ATC code level 2)	Number of detected interactions	Interaction propensity
Corticosteroids for systemic use (H02)	13	0.12
Diuretics (C03)	10	0.16
Antimetabolites (L01B)	9	0.30
Antithrombotic agents (B01)	8	0.11
Agents acting on the renin-angiotensin system (C09)	8	0.12
Antiemetics and antinauseants (A04)	8	0.07
Antigout agents (M04)	8	0.13
Alkylating agents (L01A)	6	0.13
Drugs used in diabetes (A10)	6	0.21
Antibiotics (J01)	6	0.86
Others	18	-

### Association of long-term medication before start of cancer treatment with severe toxicity

One hundred thirteen patients were available for outcome analysis with complete follow-up data (further site of treatment unknown, *n* = 4; follow-up data not completely accessible, *n* = 3; follow-up not conducted (pilot study), *n* = 16). Overall toxicity grade ≥ 3 was documented in 92 (81.4%) patients; 76 (67.3%) showed hematological toxicity grade ≥ 3 and 67 (59.3%) nonhematological toxicity grade ≥ 3 (for more details regarding toxicity see [8]).

For overall and hematological toxicity, the occurrence of rPDDI in the long-term medication before start of cancer treatment was significantly associated with grade ≥ 3 toxicity in univariate logistic regression: Patients with rPDDI exhibited an approximately fivefold risk of developing overall toxicity (OR, 5.07; *p* = 0.036; 95% Confidence Interval (CI) 1.11–23.14; toxicity in patients with rPDDI 94.1% (32/34) vs 75.9% (60/79) in patients without rPDDI) and an approximately fourfold risk of experiencing hematological toxicity (OR, 3.95; *p* = 0.010; 95% CI 1.38–11.29; hematological toxicity in patients with rPDDI 85.3% (29/34) vs 59.5% (47/79) in patients without rPDDI). However, the occurrence of rPDDI was not associated with nonhematological toxicity. Instead, nonhematological toxicity was significantly associated with the number of drugs per patient and the number of PIM drugs per patient. Corresponding details are displayed in Table 6.

**Table 6:** Univariate logistic regression of grade ≥ 3 toxicity during therapy related to risks in long-term medication (*n* = 113); distribution of CTCAE grade ≥ 3 toxicity in patients during therapy course per medication risks

	**Odds ratio** **(95% CI)**	***P* value**	**Number of patients with toxicity/number of all patients (%) with vs *without* medication risk**
**Overall toxicity**			
Number of drugs per patient	1.15 (0.98–1.34)	0.090	-
Patients with vs *without (reference)* polymedication	1.52 (0.58–3.95)	0.391	49/58 (84.5) vs 43/55 (78.2)
Number of PIM per patient	1.23 (0.71–2.13)	0.460	-
Patients with vs *without (reference)* at least one PIM	1.31 (0.51–3.39)	0.578	50/60 (83.3) vs 42/53 (79.2)
Number of rPDDI per patient	3.84 (0.97–15.31)	0.056	-
Patients with vs *without (reference)* at least one rPDDI	5.07 (1.11–23.14)	0.036	32/34 (94.1) vs 60/79 (75.9)
**Hematological toxicity**			
Number of drugs per patient	1.04 (0.93–1.16)	0.511	-
Patients with vs *without (reference)* polymedication	1.17 (0.53–2.58)	0.691	40/58 (69.0) vs 36/55 (65.5)
Number of PIM per patient	0.91 (0.60–1.36)	0.642	-
Patients with vs *without (reference)* at least one PIM	0.94 (0.43–2.08)	0.887	40/60 (66.7) vs 36/53 (67.9)
Number of rPDDI per patient	1.59 (0.90–2.80)	0.111	-
Patients with vs *without (reference)* at least one rPDDI	3.95 (1.38–11.29)	0.010	29/34 (85.3) vs 47/79 (59.5)
**Nonhematological toxicity**			
Number of drugs per patient	1.14 (1.01–1.28)	0.029	-
Patients with vs *without (reference)* polymedication	1.47 (0.69–3.12)	0.318	37/58 (63.8) vs 30/55 (54.5)
Number of PIM per patient	1.68 (1.05–2.67)	0.030	-
Patients with vs *without (reference)* at least one PIM	1.93 (0.90–4.12)	0.091	40/60 (66.7) vs 27/53 (50.9)
Number of rPDDI per patient	1.59 (0.95–2.66)	0.076	-
Patients with vs *without (reference)* at least one rPDDI	1.66 (0.72–3.87)	0.238	23/34 (67.6) vs 44/79 (55.7)

Reference: in italic; if no reference is given the variable was treated as continuous; Polymedication: ≥ 5 long-term drugs per patient

When ECOG and age were included into the model, rPDDI yielded for overall toxicity an odds ratio of 4.56 (95% CI 0.98–21.29) which was not statistically significant (*p* = 0.054) and for hematological toxicity an odds ratio of 4.51 (95% CI 1.52–13.38) remaining statistically significant (*p* = 0.007). For nonhematological toxicity, results for the number of drugs per patient and the number of PIM drugs per patient were not statistically significant in the multivariate analysis including ECOG and age (number of drugs: OR 1.1, 95% CI 0.97–1.25, *p* = 0.134; number of PIM drugs: OR 1.52, 95% CI 0.93–2.47, *p* = 0.095).

## Discussion

To the best of our knowledge, this is the first study analyzing medication risks regarding polymedication, potentially inappropriate medication as well as drug-drug interactions for a cohort of older cancer patients in a German hospital setting and evaluating their impact on therapy-associated toxicity. There is another German study investigating polymedication and its association with severe therapy-related toxicity in a German hospital setting. However, this study only investigated ovarian cancer patients and did not exclusively focus on older patients [14].

### Medication risks

In our study, medication risks were common in older patients with cancer even before initiation of cancer therapy: 52.2% of patients were exposed to polymedication, 52.9% to potentially inappropriate medication (PIM), and 30.9% to relevant potential drug-drug interactions (rPDDI). Moreover, our results suggest that medication risks may impair patient safety by leading to adverse outcomes. Relevant potential drug-drug interactions were significantly associated with severe overall and hematological toxicity.

Comparing our results with previous studies, heterogeneous definitions of polymedication, PIM or drug-drug interactions as well as differences in data collection have to be considered. Turner et al. reported a prevalence of 57% for polymedication and 15% for hyperpolymedication, which is similar to our results [4]. The detected PIM prevalence of our study lies within the range of previously reported results as well. A study by Reis et al. indicated that 48.1% of older patients with cancer used at least one PIM drug (according to 2015 Beers criteria) [6]. Concerning rPDDI, Yeoh et al. found that 55.1% of older patients with cancer are exposed to potential drug-drug interactions [1]. Because our study used the ABDA database classification system which is common in Germany but rather unknown in other countries, our results for drug-drug interactions might differ from other studies. Different interaction information systems have presented deviant listing of interactions and variant severity classifications [15].

After the start of cancer therapy, patients received additional PIM and rPDDI in our study, suggesting that the overall number of medication risks increased as well. In contrast, Karuturi et al. found a decrease of PIM prevalence in older patients after the diagnosis of breast or colorectal cancer (PIM prevalence breast cancer: pre-chemotherapy 36.6% vs 0–3 months after start of chemotherapy 27.9% vs 3–6 months after start of chemotherapy 20%) [16].

Since some PIM are required as pre-medication or supportive care medication in cancer therapy, the benefit-risk assessment of these PIM drugs in cancer patients may differ from other older patients. Nevertheless, our analysis included all PIM drugs because, regardless of its use in supportive therapy, they bear risks in older patients which physicians should be aware of. Feng et al. only reported a slight difference in prevalence when neglecting the appropriate PIM drugs for cancer patients compared to including all PIM drugs [17]. Regarding the rPDDI of the antineoplastic/supportive care medication, the results of this analysis suggest particular caution when prescribing serotonin 5-HT3 receptor antagonists due to the severity of triggered rPDDI. The two most severe interaction types were both caused by serotonin 5-HT3 receptor antagonists due to their QT prolonging properties. This might be of special concern in older patients with frequent cardiovascular diseases. Moreover, special attention is required when administering antibiotics being the drug class with the highest interaction propensity. This is particularly due to the interactions between trimethoprim and ACE inhibitors/sartans. Because both drugs/drug classes may increase potassium levels in serum, a combined use bears the risk of hyperkalemia. It should be kept in mind that the soaring use of targeted drugs in anticancer therapy with various cytochrome P450-mediated interactions may further increase the probability for relevant drug-drug interactions between antineoplastic therapy and long-term medication.

Specific multidisciplinary interventions involving oncologists, geriatricians, and clinical pharmacists should be further developed in order to appropriately address the issue of polymedication, PIM and rPDDI in older cancer patients. A pharmacist-led, individualized medication assessment reduced the average number of drug-related problems by 45.5% [18]. Further studies showing the efficacy of such interventions are urgently needed.

### Association with severe toxicity

We analyzed the association of the medication taken before start of treatment with subsequent toxicity during therapy cycles in order to see if pre-existing medication risks may influence therapy tolerance. In literature, results were not consistent regarding the association between the number of drugs and severe toxicity in older patients with cancer [5]. In line with our results, a secondary analysis did not find an association of the number of daily drugs before start of chemotherapy and overall chemotherapy-related toxicity [19]. However, Hamaker et al. detected a significant association between baseline polymedication and severe toxicity during cancer treatment of older metastatic breast cancer patients [20]. Concerning PIM, Maggiore et al. did not report any association between PIM use and overall grade ≥ 3 toxicity, being consistent with our findings [19]. Occurrence of rPDDI was significantly associated with grade ≥ 3 overall and hematological toxicity in our study. However, a study by Popa et al. indicated that potential drug-drug interactions were not associated with grade 4 hematological toxicity [21]. In contrast, grade ≥ 3 nonhematological toxicity was significantly associated with potential drug-drug interactions of higher severity (“level 1–3”) in that study. These differences might be caused by the use of different softwares for classifying the severity of potential drug-drug interactions.

### Strengths and limitations

A strength of this study is the evaluation of the risk-outcome association, being essential for assessing the clinical implications of our findings. Moreover, the investigation of two distinct time points allowed analyzing the changes in medication before and after the start of cancer therapy. In addition, this is the first study assessing PIM use of older patients with cancer via the EU(7)-PIM list. On the other hand, the study is limited by its retrospective character. The documentation of drugs in the medical records might have been incomplete, leading to an underestimation of drug use. Moreover, further information on the duration and rationale of drug use was partly missing due to the retrospective design. This might have limited the judgment of PIM. However, by selecting an explicit PIM list, only little additional data was required. A further limitation is the moderate sample size of the analysis. The results from the univariate analysis were further investigated by adjusting for the clinically relevant confounding factors ECOG and age. However, results of the multivariate analysis as well as the univariate analysis have to be interpreted with caution due to the sample size. Because of the moderate number of patients, our analysis could only account for a limited number of confounding factors. However, there might be still other potential factors which could have influenced the results. Therefore, further studies with a prospective design and a larger patient cohort are needed to corroborate our findings. Nevertheless, our results already clearly suggest the pitfalls regarding the medication of elderly cancer patients and hence underline the importance of addressing this topic for ensuring patient safety.

## Conclusion

The results of our analysis indicate that medication risks are common in older cancer patients and might be associated with the toxicity of anticancer therapies. Specific multidisciplinary interventions should be implemented to enhance patient safety in this vulnerable patient group. Apart from toxicity, other patient-relevant endpoints like hospitalization or survival could be of interest for further analyses. Registry-based trials might be useful for gaining more insight into the consequences of medication risks under real-world conditions.

## Supplementary Information

**Additional file 1: Supplement 1.** Drugs and drug classes (ATC code level 2) of patients’ long-term medication before start of cancer therapy (*n* = 136); ASS, acetylsalicylic acid; HCT, hydrochlorothiazide. **Supplement 2.** Drug classes (ATC code level 2) and individual drugs which patients received as antineoplastic agents or supportive care medication after start of cancer therapy (*n* = 128).

## Data Availability

The datasets used and/or analysed during the current study are available from the corresponding author on reasonable request.

## Abbreviations

ABDA: Federal Union of German Associations of Pharmacists (Bundesvereinigung Deutscher Apothekerverbände)
ATC: Anatomical Therapeutic Chemical
CARG: Cancer and Aging Research Group
CRASH: Chemotherapy Risk Assessment Scale for High-Age Patients
CTCAE: Common Terminology Criteria for Adverse Events
DRP: Drug-related problems
F_i_: Relative frequency of a drug or drug class being involved as interaction partner in rPDDI
IP: Interaction propensity
IQR: Interquartile range
OR: Odds ratio
OTC: Over-the-counter
PIM: Potentially inappropriate medication
PPI: Proton-pump inhibitors
P_d_: Prevalence of a drug or drug class
rPDDI: Relevant potential drug-drug interactions.
SD: Standard deviation
