# Supplementary material for: Medication risks in older patients (70 +) with cancer and their association with therapy-related toxicity
Source: BMC Geriatr. 2022 Aug 30;22:716. doi: 10.1186/s12877-022-03390-z (PMC9429305; doi:10.1186/s12877-022-03390-z)
Supplement: Supplementary file 1 — Additional file 1: Supplement 1. Drugs and drug classes (ATC code level 2) of patients’ long-term medication before start of cancer therapy (n = 136); ASS, acetylsalicylic acid; HCT, hydrochlorothiazide. Supplement 2. Drug classes (ATC code level 2) and individual drugs which patients received as antineoplastic agents or supportive care medication after start of cancer therapy (n = 128). [file 12877_2022_3390_MOESM1_ESM.docx]

**Supplements**

**Supplement 1** Drugs and drug classes (ATC code level 2) of patients’ long-term medication before start of cancer therapy (n = 136); ASS, acetylsalicylic acid; HCT, hydrochlorothiazide

| **Drug class (ATC code level 2)** | | **Number of drug prescriptions** |
| --- | --- | --- |
| Antithrombotic agents (B01) | | 70 |
| Agents acting on the renin-angiotensin system (C09) | | 65 |
| Diuretics (C03) | | 59 |
| Beta blocking agents (C07) | | 50 |
| Lipid modifying agents (C10) | | 48 |
| Drugs for acid-related disorders (A02) | | 47 |
| Thyroid therapy (H03) | | 46 |
| Analgesics (N02) | | 32 |
| Calcium channel blockers (C08) | | 30 |
| Drugs used in diabetes (A10) | | 29 |
| Others | | 207 |
| **Drug** | **Number of patients** | **Proportion of patients**  **with respective drug [%]** |
| Pantoprazole | 42 | 30.9 |
| L-thyroxine | 38 | 27.9 |
| ASS | 35 | 25.7 |
| Simvastatin | 31 | 22.8 |
| HCT | 29 | 21.3 |
| Bisoprolol | 22 | 16.2 |
| Ramipril | 22 | 16.2 |
| Amlodipine | 21 | 15.4 |
| Metoprolol | 20 | 14.7 |
| Candesartan | 17 | 12.5 |
| Metamizole | 17 | 12.5 |

**Supplement 2** Drug classes (ATC code level 2) and individual drugs which patients received as antineoplastic agents or supportive care medication after start of cancer therapy (n = 128)

| **Antineoplastic agents** |  |
| --- | --- |
| **Drug class (ATC code level 2)** | **Number of drug prescriptions** |
| Plant alkaloids and other natural products (L01C) | 76 |
| Platinum compounds (L01XA) | 54 |
| Alkylating agents (L01A) | 45 |
| Monoclonal antibodies (L01XC) | 40 |
| Antimetabolites (L01B) | 30 |
| Corticosteroids for systemic use (H02) | 29 |
| Cytotoxic antibiotics and related substances (L01D) | 29 |
| Others | 8 |
| **Drug** | **Number of patients** |
| Paclitaxel | 38 |
| Carboplatin | 37 |
| Rituximab | 33 |
| Cyclophosphamide | 30 |
| Doxorubicin | 25 |
| Vincristine | 23 |
| Prednisolone | 13 |
| Predisone | 11 |
| Oxaliplatin | 10 |
| Fluorouracil | 9 |
| Etoposide | 9 |
| Bendamustine | 8 |
| Gemcitabine | 8 |
| Cisplatin | 7 |
| Decitabine | 6 |
| Bortezomib | 5 |
| Dexamethasone | 5 |
| Methotrexate | 4 |

**Supplement 2 (continued)**

| **Supportive care medication** |  |
| --- | --- |
| **Drug class (ATC code level 2)** | **Number of drugs prescriptions** |
| Antiemetics and antinauseants (A04) | 116 |
| Antihistamines for systemic use (R06) | 71 |
| Corticosteroids for systemic use (H02) | 62 |
| Drugs for acid-related disorders (A02) | 60 |
| Antigout preparations (M04) | 49 |
| Analgesics (N02) | 35 |
| Detoxifying agents for antineoplastic treatment (V03AF) | 34 |
| Others | 38 |
| **Drug** | **Number of patients** |
| Ondansetron | 109 |
| Dexamethasone | 62 |
| Ranitidine | 52 |
| Clemastine | 50 |
| Allopurinol | 49 |
| Paracetamol | 35 |
| Mesna | 32 |
| Dimetindene | 21 |
| Calcium folinate | 11 |
